# XadA2 Adhesin Decreases Biofilm Formation and Transmission of *Xylella fastidiosa* subsp. *pauca*

**DOI:** 10.3390/insects11080473

**Published:** 2020-07-26

**Authors:** Mariana Bossi Esteves, Julia Lopes Nalin, Karla Kudlawiec, Raquel Caserta Salviatto, Tiago de Melo Sales, Anne Sicard, Rodrigo Piacentini Paes de Almeida, Alessandra Alves de Souza, João Roberto Spotti Lopes

**Affiliations:** 1 Departamento de Entomologia e Acarologia, Escola Superior de Agricultura “Luiz de Queiroz” (ESALQ), Universidade de São Paulo (USP), Piracicaba, SP 13418-900, Brazil; julia.nalin@hotmail.com (J.L.N.); kkudlawiec@usp.br (K.K.); jrslopes@usp.br (J.R.S.L.); 2 Centro de Citricultura “Sylvio Moreira”, Instituto Agronômico, Cordeirópolis, SP 13490-970, Brazil; rcsalviatto@gmail.com (R.C.S.); desouza@ccsm.br (A.A.d.S.); 3 Instituto Federal do Pará, Castanhal, Pará 68740-970, Brazil; tiago.sales@ifpa.edu.br; 4 Department of Environmental Science, Policy, and Management, University of California, Berkeley, CA 94720-3114, USA; anne.sicard@berkeley.edu (A.S.); rodrigoalmeida@berkeley.edu (R.P.P.d.A.)

**Keywords:** sharpshooter, vector-borne, attachment, bacterium, blocking transmission

## Abstract

*Xylella fastidiosa* is a vector-borne bacterium that causes diseases in many plants of economic interest. The bacterium–vector initial interactions involve bacterial membrane-bound adhesins that mediate cell attachment to the foregut of insect vectors. We investigated the role of the afimbrial adhesin XadA2 in the binding and biofilm formation of *X. fastidiosa* subsp. *pauca* to vector surfaces in vitro, as well as its potential to disrupt pathogen transmission. We showed that XadA2 has binding affinity for polysaccharides on sharpshooter hindwings, used as a proxy for the interactions between *X. fastidiosa* and vectors. When in a medium without carbon sources, the bacterium used wing components, likely chitin, as a source of nutrients and formed a biofilm on the wing surface. There was a significant reduction in *X. fastidiosa* biofilm formation and cell aggregation on vector wings in competition assays with XadA2 or its specific antibody (anti-XadA2). Finally, pathogen acquisition and transmission to plant were significantly reduced when the vectors acquired *X. fastidiosa* from an artificial diet supplemented with anti-XadA2. These results show that XadA2 is important in mediating bacterial colonization in the insect and that it could be used as a target for blocking *X. fastidiosa* transmission.

## 1. Introduction

Vector-borne bacterial plant pathogens are responsible for some of the most important emerging plant diseases today [1]. *Xylella fastidiosa* is a bacterium that causes several diseases in agricultural crops of economic interest, including citrus variegated chlorosis (CVC), Pierce’s disease (PD) of grapevines and olive quick decline syndrome (OQDS) [2,3]. It is naturally disseminated among host plants by xylem-sap feeding insects, known as sharpshooter leafhoppers (Hemiptera: Cicadellidae: Cicadellinae) and spittlebugs (Hemiptera: Cercopoidea) [4].

Previous studies have shown that initial interactions of *X. fastidiosa* with the foregut of vectors, the retention site of the pathogen, are mediated by adhesion proteins on the bacterial cell surface, which are classified as fimbrial and afimbrial adhesins [5]. Adhesins have a fundamental role in *X. fastidiosa*–insect vector interactions. Killiny and Almeida [5,6] demonstrated that the adhesins HxfA, HxfB and FimA are fundamental for initial adhesion and maintenance of the biofilm in the vector foregut, since mutants for these adhesins were less efficiently acquired and retained by insect vectors. Furthermore, a significant reduction in transmission efficiency to plants was observed when *X. fastidiosa* cells were acquired by the insect vector from artificial diets containing peptides derived from proteins involved in bacterial colonization of vectors, probably due to competition between bacterial cells and peptides for the foregut binding sites [7,8].

For *X. fastidiosa* subsp. *pauca* strains that cause CVC, the role of adhesins in vector foregut colonization has not been studied. Studies have shown that afimbrial adhesins such as XadA1 and XadA2 seem to be important in different steps of biofilm formation [9]. XadA2 exhibits Hia domains, which are also found in the Hsf protein of *Haemophilus influenzae* and allow adhesion to mammalian epithelial cells [10]. In *X. fastidiosa* subsp. *pauca,* XadA2 shows other domains with putative chitin adhesive function, for instance, the Hep-Hag and hemagglutinin domains. In addition, analysis of XadA2 revealed the presence of motifs that are unique to proteins from *X. fastidiosa* [9], suggesting a specific role of XadA2 in this bacterium life cycle in the host plant and/or insect vector.

A major difficulty for studying *X. fastidiosa* adhesion and biofilm formation in vectors is that the foregut is located inside the head of the insect, requiring detailed histological sections for its microscopic visualization. Considering that the whole exoskeleton of the insect consists of structural polysaccharides such as chitin, similar to the cuticular surface of the foregut, insect wings have been used as an experimental binding surface for such studies [5,11]. Therefore, this work evaluated the role of XadA2 in *X. fastidiosa* subsp. *pauca* biofilm formation on insect vector wings as the adhesion substrate. Additionally, we demonstrated the impact of XadA2 on the acquisition and transmission efficiency of *X. fastidiosa* by sharpshooters.

## 2. Materials and Methods

### 2.1. Insects

The sharpshooter species *Bucephalonia xanthophis* (Berg), *Macugonalia leucomelas* (Walker) and *Sibovia sagata* (Signoret) were collected on shrubs and trees [*Lagerstroemia indica* L. (Lythraceae), *Duranta repens* L. (Verbenaceae), *Vernonia condensata* (Asteraceae), and *Hibiscus* spp. (Malvaceae)] in Piracicaba, São Paulo state, Brazil. Approximately 50–60 adult individuals of each species were placed inside screened cages with *V. condensata* or *Ocimum basilicum* (Lamiaceae) plants for rearing healthy individuals of *B. xanthophis* or *M. leucomelas* and *S. sagata*, respectively, as described previously [12]. The cages were kept in a greenhouse at 25 ± 5 °C.

### 2.2. Xylella fastidiosa Strains

*X. fastidiosa* subsp. *pauca* strains 9a5c [13] and 11399 were used for the experiments. Strain 11399 was modified with green fluorescent protein (GFP) [14] and named *X. fastidiosa*-GFP. Periwinkle wilt gelrite (PWG) medium [15] was used for cell growth. PWG supplemented with kanamycin (50 μg/mL) was used for *X. fastidiosa*-GFP.

### 2.3. Expression and Purification of XadA2

The expression and purification of XadA2 was performed using the vector constructed by Caserta et al. [9]. The protein was induced using 1 mM isopropyl-β-d-thiogalactopyranoside (IPTG) in a 100 mL culture incubated for 2 h at 37 °C. The cells were collected by centrifugation at 4.000×g for 20 min at 8 °C, as previously described [9]. The expression pattern of XadA2 was checked by SDS-PAGE 12% stained with Coomassie brilliant blue. The protein was purified using an immobilized metal affinity chromatography (IMAC) column packed with 1.0 mL of nickel-nitrilotriacetic acid (Ni-NTA) resin. Bound proteins were eluted with 4 mL of 200 mM imidazole in 50 mM Tris (pH 7.5), 300 mM NaCl. Aliquots were used to estimate the total protein concentration (Bradford assay) and were analyzed by SDS-PAGE. As the samples were eluted in buffer containing imidazole, a reagent that may influence the results of the bioassays, a chemical removal process was performed using the Amicon^®^ Ultra-0.5 filter devices (Millipore, Burlington, MA, USA) column kit following the manufacturer’s recommendations.

### 2.4. XadA2 Affinity for Chitin and Cellulose

This in vitro assay followed the methodology described by Labroussaa el al. [7]. Colloidal chitin and cellulose were resuspended in buffer (KH_2_PO_4_ 2 mM, Na_2_HPO_4_ 8 mM, KCl 2 mM, pH 7). Then, each polysaccharide (1 mg/mL) was placed in microtubes with 50 μg/mL XadA2 protein, and bovine serum albumin (BSA) was used as a control. In order to normalize the results, each protein (XadA2 e BSA) was placed in microtubes at a concentration of 50 μg/mL without the presence of the polysaccharides. All samples were incubated for 1 h at 4 °C, 60 rpm, and then centrifuged at 13.000 rpm for 3 min. The supernatant was removed for quantification of the remaining protein on Varioskan LUX Multimode Microplate reader equipment (Thermo Fisher Scientific, Waltham, MA, USA). This assay was performed with three replicates in each block (*n* = 6).

### 2.5. Cell Aggregation Assay

*X. fastidiosa*-GFP was grown on solid PWG for 7 days, when the cells were transferred to the liquid *X. fastidiosa* medium (XFM) [16]. An aliquot was taken to quantify the optical density (OD) at time zero. Then, 3 mL of the cell suspension and the synthetic XadA2 protein (10 μg/mL) were added to a Falcon tube. Suspensions without the XadA2 protein were used as control. From each tube, every hour, over a 6-h period, 150 μL aliquots of the supernatant were taken without disturbing the tubes. These aliquots were used to determine OD at 600. This experiment was conducted three times, in independent biological replicates.

### 2.6. Biofilm Formation on Sharpshooter Wings

For this experiment, as well as in previous studies [5,11], sharpshooter hindwings were used for simulating attachment of *X. fastidiosa* to the vector foregut. The biofilm formation experiments were initially performed using three species of sharpshooters: *B. xanthophis*, *M. leucomelas* and *S. sagata*. The wings disinfection process was performed as follows: ethanol 70% for 2 min, liquid bleach (active chlorine 2.5%) for 2 min and three rinses in autoclaved water for 2 min each. The wings were placed separately according to the species in microtubes containing XFM medium liquid, depleted of their carbon sources (trisodium citrate and disodium succinate), and then known XFM-Δ, with *X. fastidiosa*-GFP resuspended at OD_600_ = 0.6. For each species of sharpshooter, 10 wings were used. Three wings of each species were incubated with XFM-Δ only (i.e., without *X. fastidiosa* cells) as controls. The wings were incubated for 10 days at 28 °C. After incubation, they were rinsed with distilled water to remove cells that were not adhered. The wing biofilms were evaluated using a fluorescent stereoscopic microscope with a GFP filter (MVX10 Olympus Life Science, Beijing, China) with 4× magnification. Controls of each species were evaluated for autofluorescence emission by wing structures. The images obtained were analyzed using the Quant software v.1.0.0.22 [17].

### 2.7. Use of Chitin as a Carbon Source

It was previously shown that *X. fastidiosa* subsp. *fastidiosa* reached larger populations in the presence of structural polysaccharides of hosts, such as chitin and pectin, in the cell culture medium [18,19], this assay used XFM-Δ with and without a chitinous surface to compare biofilm formation and bacterial population by *X. fastidiosa* subsp. *pauca*. The wings and the coverslips (used as controls) were sterilized as previously described. *X. fastidiosa*-GFP cells were grown on PWG with kanamycin (50 μg/mL) and resuspended in XFM-Δ at an OD_600_ = 0.6. The medium containing the bacterial suspension and 10 wings were added in a microtube. In another microtube, 10 pieces of coverslips were added to the bacterial suspension. The control consisted of three wings and three coverslips that were placed in separate microtubes containing only XFM-Δ. All treatments were incubated for 10 days at 28 °C. After incubation, the biotic (wings) and abiotic (coverslips) surfaces were rinsed twice with distilled water, and five samples representative of each treatment were analyzed by a fluorescent stereoscopic microscope as described previously. The other five representative samples from the treatments containing the bacterium were plated on PWG with kanamycin (50 μg/mL) in order to quantify the cells that were alive at the end of the incubation period (10 days). The counting of the colony forming units (CFU) was performed with 4× magnification with a fluorescent stereoscopic microscope.

### 2.8. Saturation of Insect Wing Binding Sites with XadA2

Hindwings of *M. leucomelas* were disinfected as described above, and then used in the following treatments: I) wings + 4 h incubation with 10 μg/mL of XadA2 + addition of XFM-Δ and *X. fastidiosa*-GFP (OD 600 nm = 1); II) wings + XFM-Δ *X. fastidiosa*-GFP (OD_600_ = 1) (positive control) and III) wings + XFM-Δ without *X. fastidiosa*-GFP (negative control). Ten replicates were performed for each treatment and incubated for 10 days at 28 °C for bacterial growth. Subsequently the wings were washed with distilled water, evaluated by a fluorescence stereoscopic microscope and analyzed as described above.

### 2.9. Blocking of X. fastidiosa Adhesion on Insect Wings by Anti-XadA2 Antibody

Hindwings of *M. leucomelas* were used as the adhesion substrate and disinfected as described above. The polyclonal antibody was provided by Caserta et al. [9]. *X. fastidiosa*-GFP was previously grown for 7 days on PWG with kanamycin and resuspended in XFM-Δ until OD_600_ = 0.6. Half of the cell suspension (approximately 500 μL) was placed in a microtube containing only the wings, representing the positive control. The anti-XadA2 antibody was added at a concentration of 2:1000 to the remaining bacterial suspension and wings. Ten replicates were performed for each treatment. Three wings incubated only with the XFM-Δ were used as negative control. After 10 days, the wings were washed and quantified as described before.

### 2.10. Blocking of Vector Acquisition and Transmission of X. fastidiosa by Anti-XadA2 Antibody

The *X. fastidiosa* 9a5c strain was grown in XFM solid for 10–12 days at 28 °C. The resulting colonies were resuspended in an artificial diet [18], with the bacterial concentration adjusted to 10^8^ CFU/mL (OD_600_ = 0.6). The diet containing *X. fastidiosa* cells was used for in vitro acquisition by the sharpshooter *M. leucomelas* through a membrane feeding system, which was assembled as described previously [11]. The experimental unit consisted of a membrane sachet with 35 μL of diet and one adult insect (previously starved for 1 h) per sachet. The experiment was repeated three times with 20 replicates (*n* = 60) of the treatments: I) diet with *X. fastidiosa*; and II) diet with *X. fastidiosa* and anti-XadA2 antibody (2:1000). As a negative control in each block, 10 insects were fed on sachets containing the artificial diet without *X. fastidiosa*. After the acquisition access period (AAP) of 6 h on the diet sachets, the insects from each treatment were divided into pairs and placed on healthy plants of *Catharanthus roseus* L. (Apocynaceae; periwinkle, an experimental host) for an inoculation access period (IAP) of 72 h. The insects were confined on the youngest leaves (4–5) of the test plants by using hinged plastic cages (5.5 × 4 cm) (model G320, Gary Plastic Packaging Corp, Bronx, NY, USA) containing a circular aperture with an anti-aphid screen for ventilation. Both the AAP and IAP were performed in a climatized room at 25 ± 2 °C under fluorescent light (150W, photophase of 14 h).

To estimate *X. fastidiosa* acquisition efficiency, sharpshooter heads were removed after the IAP and individually submitted to DNA extraction as described by [20], followed by real-time polymerase chain reaction (qPCR) [11] using the XF16Sf/XF16Sr primer set [21]. Only samples with a cycle threshold (CT) value ≤ 30 were considered positive, according to a standard curve based on serial dilutions of *X. fastidiosa* DNA (extracted from bacterial colonies) in whole DNA from heads of uninfected sharpshooters [12].

Transmission efficiency was estimated by determining the proportion of *X. fastidiosa*-infected *C. roseus* at 60 days after the IAP. About 4–5 leaves from each plant were sampled for DNA extraction [22]. The PCR for *X. fastidiosa* detection was done using the RST31/RST33 primer set [23].

### 2.11. Toxicity Assay with Anti-XadA2 Antibody

The effect of the antibody on the survival of *M. leucomelas* was evaluated under the same membrane feeding system and conditions described in the previous assay. After being starved for 1 h, 1-week-old sharpshooter adults were individually confined on artificial diet containing *X. fastidiosa* cells (strain 9a5c) (OD_600_ = 0.4), and anti-XadA2 at the following dilutions: 0.5:1000; 1:1000; 2:1000; 4:1000; or 20:1000. For each antibody dilution, 25 insects were tested. As negative control, 10 sharpshooters were confined on artificial diets containing only *X. fastidiosa* cells. After 6 h on the artificial diets, the insects were transferred to *V. condensata* plants and their survival was evaluated daily for 96 h.

### 2.12. Statistical Analysis

In relation to the in vitro assay of XadA2 protein’s affinity for carbohydrates, the values obtained did not meet the assumptions required for conventional analysis of variance; therefore, generalized linear models (GLM) were used, applying the chi-square test (*p* < 0.05) to verify the differences between the treatments and the Tukey test (*p* < 0.05) for the comparison of means, with the package multcomp [24]. In the experiment using chitin as the carbon source, the fluorescence (proportions) and bacterial population (CFU/mL) data were submitted to a Student’s *t*-test (*p* < 0.05). A repeated-measure one-way ANOVA was used, with the package Ez 4.4-0 [25] for analysis of the cell aggregation assay, where the values obtained between the treatments throughout evaluation times (0, 60, 120, 180, 240, 300 and 360 min) were compared, considering time as a repeated measure and the presence or absence of XadA2 as a fixed effect. For the fluorescent area determined in the experiments of biofilm formation on sharpshooter wings, saturation of the wing binding sites by synthetic XadA2 and *X. fastidiosa* adhesion blocking on wings by the anti-XadA2 antibody were analyzed as proportion data. The transformed data were submitted to the analysis of variance and the means were compared by the Tukey test (*p* < 0.05). For the experiment of blocking vector acquisition and transmission of *X. fastidiosa* by the anti-XadA2 antibody, 2 × 2 containment tables were created with the number of positives and negatives provided by the treatments and submitted to a Fisher’s exact test (*p* < 0.05). For the toxicity assay, the G-rho family of tests [26] was applied to compare survival curves using the statistical package Survival 2.41-3 [27]. For all the experiments, the data were submitted to the Shapiro–Wilk test to verify the normality and Bartlett test to verify the homogeneity of variances. When data obtained were proportions, these were transformed into Arcsine √x. All statistical analysis was performed in R version 3.4.4 [28] and the R Studio computer software version 1.1.447 [29].

## 3. Results

### 3.1. XadA2 Has Affinity for Cellulose and Chitin, and No Influence on X. fastidiosa Aggregation

**Figure 1:**
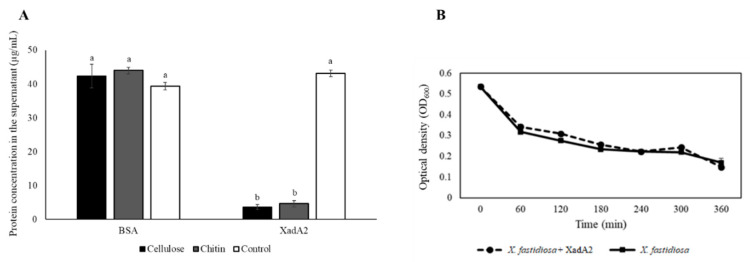
Affinity of *Xylella fastidiosa* XadA2 adhesin to polysaccharides and its influence on the bacterial aggregation capacity. (**A**) Mean concentration (±SEM, *n* = 6) of the synthetic XadA2 and BSA proteins remaining in the supernatant after incubation with cellulose and chitin, and with the buffer without polysaccharides (control). Means followed by equal letters, for each protein, did not differ statistically by the chi-square test (*p* < 0.0001). (**B**) Mean values (±SEM, *n* = 3) of absorbance when *X. fastidiosa*-GFP was incubated in *X. fastidiosa* medium (XFM) with or without synthetic XadA2.

After incubation of XadA2 with cellulose or chitin, the remaining protein concentration in the supernatant was one order of magnitude lower than the concentration observed with buffer alone (43.15 μg/mL) (*X^2^* = 530.69; d.f. = 5; *p* < 0.0001) (Figure 1A), indicating that most of the XadA2 molecules adhered to these polysaccharides. When BSA was used as a control, the remaining protein concentrations for the substrates chitin (44.09 μg/mL) and cellulose (42.36 μg/mL) were similar to that observed in the buffer alone, indicating no affinity of BSA for these substrates. No difference was found in absorbance values over the 6-hour evaluation period when *X. fastidiosa*-GFP was incubated in XFM with or without XadA2 (F = 2,679; d.f. = 1; *p* = 0.153) (Figure 1B), demonstrating that the addition of this protein did not influence the formation of cell aggregates by the bacterium.

### 3.2. X. fastidiosa Biofilm Formation on Wings is Insect Species Dependent

Wings of the three sharpshooter species exhibited autofluorescence, mainly on the site of insertion into the thorax region (Figure S1). *B. xanthophis* had a larger area of autofluorescence (2.52% ± 0.8) than *M. leucomelas* (0.95 ± 0.23%) and *S. sagata* (0.26 ± 0.13%) (F = 5.62, d.f = 2, *p* = 0.018). Although present, the autofluorescence emitted by the wing did not interfere with the visualization of the biofilm, since the software used was able to distinguish the different intensities of fluorescence in the wings. The images also showed that the three sharpshooter species allowed the formation of an *X. fastidiosa*-GFP biofilm, but with differences among them (Figure S2). The areas of fluorescence emitted by the bacterial biofilm on *M. leucomelas* wings were larger (20.96% ± 4.86) than on *B. xanthophis* (6.42 ± 2.71%) and *S. sagata* (5.15 ± 1.35%) (F = 7.88; d.f = 2; *p* = 0.006).

### 3.3. Insect Wings Support X. fastidiosa Population Growth

In XFM-Δ, the bacterium showed more extensive biofilm formation when incubated on *M. leucomelas* wings than on coverslip fragments (Figure S2). The fluorescent area on wings (16.26 ± 5.5%) was higher than on coverslips (2 ± 0.8%) (T = 3.11; d.f = 5.09; *p* = 0.025). Autofluorescence areas on wings and coverslips were estimated as 0.63 ± 0.1% and 0.07 ± 0.02%, respectively, based on the fluorescence observed in the negative controls (i.e., biotic and abiotic surfaces exposed only to the XFM-Δ medium without bacterial cells) (T = 6.83; d.f. = 3.63; *p* = 0.003). A higher population of *X. fastidiosa*-GFP cells was observed when the bacteria were incubated for 10 days on the wings (2.4 × 10^4^ CFU/mL) than on the coverslip fragments (3.9 × 10^3^ CFU/mL) (T = 5.83; d.f = 5.51; *p* = 0.001). This suggests that the bacterium was able to use polysaccharides present on the wing as a source of nutrients.

### 3.4. XadA2 Blocks X. fastidiosa Adhesion to Insect Wings

**Figure 2:**
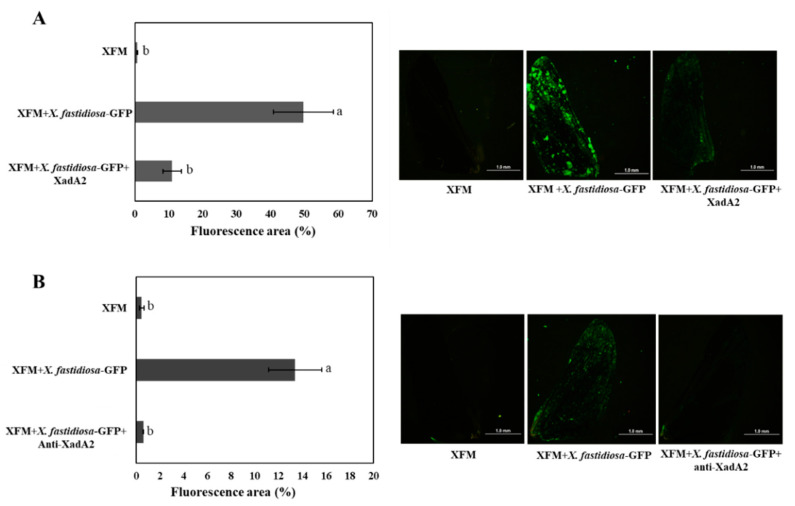
Use of *Xylella fastidiosa* XadA2 protein and specific antibody anti-XadA2 to block biofilm formation on *Macugonalia leucomelas* hindwings. (**A**) XadA2 as a competitor for insect binding sites on wings. (**B**) Use of specific antibody for the inactivation of *X. fastidiosa* XadA2. The graphics show the mean percentage (±SEM; *n* = 10) of fluorescent areas. Means followed by the same letters do not differ by Tukey’s test (*p* < 0.05). The images were obtained by a stereoscopic microscope with a GFP filter (MVX10 Olympus Olympus Life Science) with 4× magnification and show the wing autofluorescence and fluorescence areas resulting from biofilm formation by *X. fastidiosa*-GFP on wings submitted to the different treatments.

Fewer bacterial aggregates were observed on sharpshooter wings that were incubated for 4 h with XadA2 before being placed in XFM-Δ medium containing *X. fastidiosa*-GFP, in comparation with wings that were incubated with the bacterial cell suspension without previous exposure to the protein (positive control) (Figure 2A). A more fluorescent area (49.74 ± 8.6%) resulting from biofilm formation by *X. fastidiosa*-GFP was quantified on wings not exposed to XadA2 (positive control) than on wings that were first saturated with the protein and then exposed to the bacterial suspension (11 ± 2.7%) (Figure 2A) (F = 17.03; d.f = 2; *p* < 0.001). Moreover, there was no statistical difference between the treatment containing XadA2 and the negative control containing XFM medium only. Thus, these results indicate that XadA2 reduces *X. fastidiosa* attachment to vector wings.

### 3.5. X. fastidiosa Adhesion is Blocked by a XadA2 Antibody

Less biofilm formation was observed on sharpshooter wings in XFM-Δ medium with *X. fastidiosa*-GFP and anti-XadA2 antibody than on wings incubated in medium containing only *X. fastidiosa*-GFP (Figure 2B). The fluorescence area was smaller on wings of the first treatment (with anti-XadA2) (0.61 ± 0.2%) than those incubated in XFM-Δ medium only with *X. fastidiosa*-GFP (positive control) (13.4 ± 2.25%) (*X^2^* = 16.343, d.f = 2; *p* < 0.001). The treatment with the antibody did not differ statistically from the negative control (wings incubated in XFM-Δ without *X. fastidiosa*) regarding the autofluorescent area (Figure 2B).

### 3.6. XadA2 Reduces Vector Transmission of X. fastidiosa

**Figure 3:**
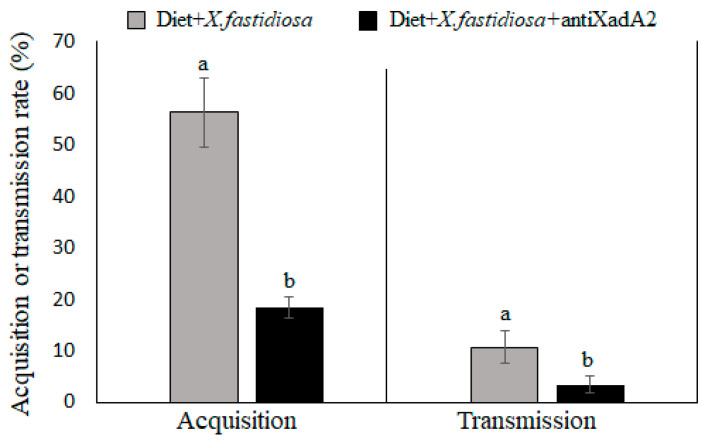
Effect of antibody against XadA2 adhesin (anti-XadA2) on the acquisition and transmission efficiencies of *Xylella fastidiosa* by *Macugonalia leucomelas* from an artificial diet in a membrane feeding system. Efficiency rates are expressed by the mean percentage of insects that acquired (±SEM; *n* = 60) or transmitted (±SEM; *n* = 30) the bacterium. Means with different letters for a same parameter (acquisition or transmission) differ statistically by Fisher’s exact test (*p* < 0.05).

The rate of *X. fastidiosa* acquisition and transmission by *M. leucomelas* was significantly affected when anti-XadA2 was added to the artificial diet containing bacterial cells. The acquisition efficiency, based on the percentage of insects qPCR-positive for *X. fastidiosa* at the end of the IAP, was about three times lower for sharpshooters fed on the diet with *X. fastidiosa* cells and the antibody (18.4 ± 2%) than for insects fed on diets containing only the bacterial cells (56.29 ± 6.7%) (Figure 3) (OR = 5.74; 95% CI = 3.21; *p* = 1.8 × 10^−8^). Likewise, a lower transmission rate per individual was estimated after acquisition of the pathogen from a diet with the antibody (3.42 ± 1.7%) in relation to a diet without it (10.67 ± 3.2%) (Figure 3) (OR = 3.97; 95% CI = 0.69; *p* = 0.024). *X. fastidiosa* was not detected in insects fed on the diet free of bacterial cells (negative control) and in the test plants exposed to these insects in the transmission assay. In addition, anti-XadA2 showed no toxic effect on the sharpshooter *M. leucomelas* (Table S1) because no significant difference in insect survival was found for any of the five antibody concentrations tested and the negative control (without antibody) over the 96-h observation period after feeding on the artificial diets (X^2^ = 4; d.f = 5; *p* = 0.546).

## 4. Discussion

The success of *X. fastidiosa* transmission by insects depends on the ability of the bacterium to colonize the cuticular lining of the foregut of vectors [30]. We explored the possibility of interfering with the initial *X. fastidiosa* subsp. *pauca* attachment to the insect vector, assuming that adhesion proteins mediate this interaction and are fundamental for the beginning of bacterial colonization, as has been demonstrated for *X. fastidiosa* subsp. *fastidiosa* [7,8]. Therefore, we investigated the role of the afimbrial adhesin XadA2 on biofilm formation of *X. fastidiosa* subsp. *pauca* on surfaces of sharpshooter vectors, and as a target for blocking cells adhesion and vector transmission to plants.

The XadA2 protein belongs to the group of trimeric autotransporter proteins of cell surfaces of Gram-negative bacteria, which mediate bacterial adhesion in host and in cell matrix proteins [31]. The region of the afimbrial protein XadA2, which comprises an antigenic fragment of 34 kDa used in this work, also shows a high degree of similarity with chitin binding proteins (CBPs) of arthropods and humans [32,33]. For the affinity assay of the XadA2 protein with polysaccharides, the most abundant polysaccharide of the insect exoskeleton, chitin, and the main component of xylem cell walls, cellulose, were chosen. We showed that the XadA2 used in this work had high binding affinity for these two host polysaccharides, since a low concentration of the remaining protein was found in the supernatant following incubation of the protein with these carbohydrates. A series of experiments carried out by Killiny and Almeida [5] showed that *X. fastidiosa* hemagglutinin-like HxfA and HxfB from subsp. *fastidiosa* of grapevines have binding affinity to chitin present on the cuticle surface of the sharpshooters and are fundamental for the initiation of biofilm formation in the vector. Although XadA2 showed higher expression intensity in the later phases of biofilm formation [9,34], its role does not appear to be related to bacterium aggregation. The addition of XadA2 in a liquid culture of *X. fastidiosa* in XFM did not increase the formation of cell aggregates, suggesting that cell-to-cell cohesion is not influenced by XadA2.

Here, sharpshooter hindwings were used as an adhesion surface to study the biofilm formation on the chitinous surface of the insect vector (similarly to the work presented in 5), since the foregut and the wings are both exocuticle and thought to have a similar composition (chitin, proteins, as well as other polysaccharides) [35]. In addition, the wings are external structures and, unlike the foregut, do not require complex histology for microscope observations. It was observed that a biofilm was formed on the wings of the three species tested, *B. xanthophis*, *M. leucomelas* and *S. sagata*. More biofilm formation was observed in *M. leucomelas*, which transmitted *X. fastidiosa* subsp. *pauca* with higher efficiency than *B. xanthophis* [36] and *S. sagata* [12]. Although differences in transmission rates may be related to several factors [37], it is tempting to speculate and propose that biofilm formation on the chitinous surface of the foregut, which is the natural retention site of *X. fastidiosa*, may occur more easily in *M. leucomelas*, contributing to the higher efficiency of this sharpshooter as a vector.

We also verified that *X. fastidiosa* subsp. *pauca* can use the chitin present in the wings of the sharpshooters as a carbon source. The use of chitin as a sole carbon source had previously been demonstrated for an *X. fastidiosa* subsp. *fastidiosa* [19,38]. We showed that the adhesion and survival of bacterial cells on an abiotic surface (glass coverslips) was lower when incubating in XFM medium without a carbon source. The opposite was observed when sharpshooter wings (a source of carbon), instead of coverslips, were added to the bacterial suspension. Similar results were obtained by Killiny et al. [38], who observed a higher *X. fastidiosa* population and amount of biofilm, as well as a more adhesive phenotype, when chitin was added to a modified XFM medium.

Two different methods were used in this work to block *X. fastidiosa* biofilm formation on sharpshooter wings. The first one was based on competition for binding sites on the wing chitinous surface with the addition of synthetic XadA2, and the second involved inhibition of bacterial adhesion by adding a specific antibody against the *X. fastidiosa* XadA2 membrane protein. We did not perform assays with a XadA2 mutant strain because, like most other efforts to generate knockout mutants for *X. fastidiosa* subsp. *pauca*, we were not able to generate a mutant of this pathogenic strain in the laboratory [39]. Both methods resulted in a significant decrease in biofilm formation on the wings, although the blocking mechanisms are most likely different. The results of the competition bioassay using XadA2 suggested the presence of specific binding sites on the insect wing. Wing surface polysaccharides were already saturated with the synthetic protein when *X. fastidiosa* cells were incubated with the wings, resulting in lower bacterial adhesion and consequently less biofilm formation. When the specific antibody against the XadA2 protein was used as a competitor molecule, a drastic reduction in biofilm aggregates was also observed. In this case, the specific anti-XadA2 antibody presumably bound to XadA2 proteins present on the bacterium membrane and prevented cells from binding to the wing and initiating biofilm formation.

The anti-XadA2 antibody was then employed to determine whether blocking *X. fastidiosa* XadA2 could impact acquisition and/or transmission by the sharpshooter vector, *M. leucomelas,* in a bioassay using an artificial diet for the ingestion of bacterial cells suspensions with or without the antibody. A three-fold reduction in both acquisition and transmission efficiencies was observed when the insects were fed on the diet containing *X. fastidiosa* suspension with the antibody in comparison with insects fed on a diet containing only the bacterial cells. When extrapolated to a field situation, this level of reduction in vector acquisition and transmission efficiencies could have a major impact on disease epidemiology, with a significant decrease in the rates of disease spread, due to lower incidences of sharpshooters carrying the pathogen and of new plants becoming infected over time. Competition assays aiming at blocking *X. fastidiosa* transmission by vectors were previously performed with the subspecies *fastidiosa* from grapes [7,8]. Killiny et al. [38] used lectins to saturate the receptors on the cuticular surface of the vector foregut, and carbohydrates and antibodies to bind to *X. fastidiosa* membrane proteins. In all approaches, vector transmission to plants was reduced. Later, Labroussaa et al. [7] developed a set of recombinant peptides with chitin affinity, which also reduced the vector transmission of *X. fastidiosa* to plants, indicating that chitin or a chitin-like substrate on the vector foregut surface is a potential target for blocking *X. fastidiosa* adhesion.

## 5. Conclusions

In summary, we showed that the adhesin XadA2 has an important role in the binding and biofilm formation of *X. fastidiosa* subsp. *pauca* to vector surfaces and that it can be used as a target for disrupting the transmission of this important pathogen. Disease spread control methods based on blocking vector transmission represent novel and promising tools that could be integrated into the current management of diseases caused by *X. fastidiosa*. More studies involving vector binding inhibitory molecules such as synthetic adhesins and their antibodies should be performed. XadA2 may be an effective target for blocking the transmission of other subspecies or strains of *X. fastidiosa*, since this membrane protein is conserved among them.

## Supplementary Materials

The following are available online at https://www.mdpi.com/2075-4450/11/8/473/s1, Figure S1: Wings of sharpshooters: *Bucephalogonia xanthophis*, *Macugonalia leucomelas* and *Sibovia sagata* after incubation in XFM-Δ medium with and without *Xylella fastidiosa* modified with the *green fluorescent protein* (GFP) gene, Figure S2: Fluorescence of the biofilm formed by *Xylella fastidiosa* fused to the *green fluorescent protein* (GFP), after 10 days of incubation at 28 °C in XFM-Δ medium containing as adhesion substrate biotic (*Macugonalia leucomelas* wing) and abiotic (coverslip) surfaces, Table S1: Mortality rates of adults of the sharpshooter *Macugonalia leucomelas* after feeding for 6 h on artificial diets with cell suspensions of *Xylella fastidiosa*, supplemented with increasing concentrations of the antibody against XadA2 (anti-XadA2).
