# Supplementary material for: XadA2 Adhesin Decreases Biofilm Formation and Transmission of Xylella fastidiosa subsp. pauca
Source: Insects. 2020 Jul 26;11(8):473. doi: 10.3390/insects11080473 (PMC7469142; doi:10.3390/insects11080473)
Supplement: Supplementary file 1 [file insects-11-00473-s001.pdf]

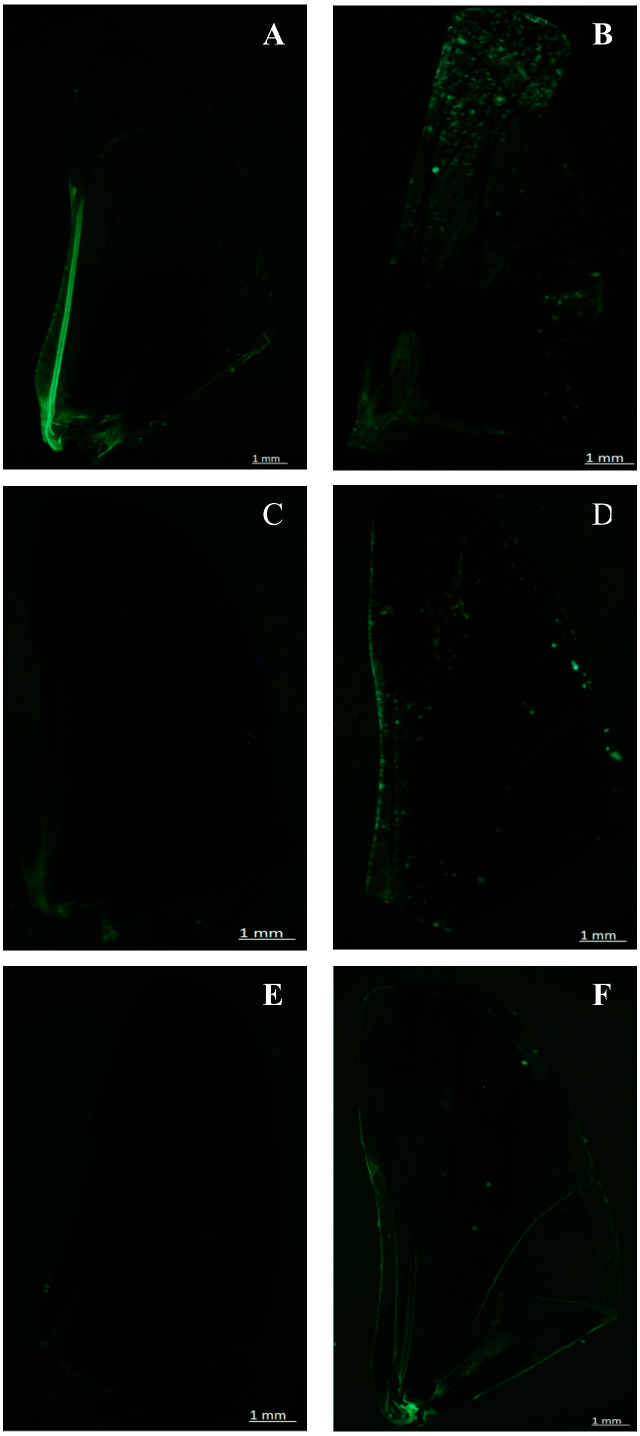

Supplementary Figure 1. Sharpshooter wings: *Bucephalogonia xanthophis*, *Macugonalia leucomelas* and *Sibovia sagata* after incubation in XFM-Δ medium with and without *Xylella fastidiosa* modified with the *green fluorescent protein* (GFP) gene. A, C and E represent the autofluorescence of the wings of *B. xanthophis*, *M. leucomelas* and *S. sagata*, respectively, which were incubated only with Xfm medium. B, D and F show the biofilm formed by *X. fastidiosa*-GFP on *B. xanthophis*, *M. leucomelas* and *S. sagata* wings, respectively. Images obtained with stereoscopic microscope with a GFP filter (Olympus MVX10) in 4x magnification.

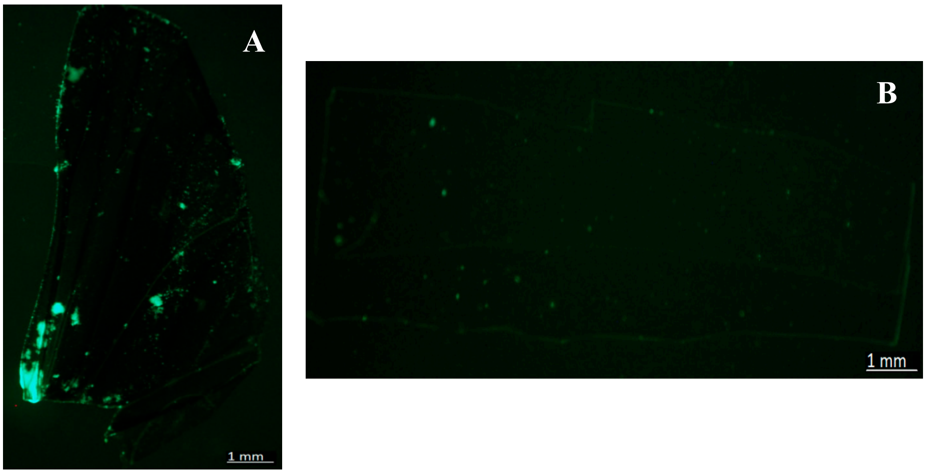

Supplementary Figure 2. Fluorescence of the biofilm formed by *Xylella fastidiosa* fused to the *green fluorescent protein* (GFP), after 10 days of incubation at 28 °C in XFM-Δ medium containing as adhesion substrate biotic (*Macugonalia leucomelas* wing) (A) and abiotic (coverslip) (B) surfaces. The images were obtained by stereoscopic microscope with a GFP filter (Olympus MVX10) in 4x magnification.

	**Time after feeding (h)**							
**Treatment (antibody concentration)**	**24**		48		72		96	
Diet + *X.fastidiosa* (without antibody)	1/10[a]	10[b]	1/10	10	1/10	10	2/10	20
Diet + *X.fastidiosa* + anti-XadA2 (0.5:1000)	1/25	4	1/25	4	1/25	4	3/25	12
Diet + *X.fastidiosa* + anti-XadA2 (1:1000)	1/25	4	2/25	8	2/25	8	5/25	20
Diet + *X.fastidiosa* + anti-XadA2 (2:1000)	1/25	4	3/25	12	3/25	12	5/25	20
Diet + *X.fastidiosa* + anti-XadA2 (4:1000)	1/25	6	1/25	4	2/25	8	3/25	12
Diet + *X.fastidiosa* + anti-XadA2 (20:1000)	0/25	0	0/25	0	0/25	0	1/25	4

[a]Number of dead insects / number of insects evaluated. [b]Percent mortality (%).

Supplementary table S1. Mortality rates of adults of the sharpshooter *Macugonalia leucomelas* after feeding for 6 h on artificial diets with cell suspensions of *Xylella fastidiosa*, supplemented with increasing concentrations of the antibody against XadA2 (anti-XadA2). There was no statistical difference in mortality rates among the treatments by the survival curves analysis in the times using G-rho family of tests ($P = 0.546$).
